# Self-application of aminoglycoside-based creams to treat cutaneous leishmaniasis in travelers

**DOI:** 10.1371/journal.pntd.0011492

**Published:** 2023-08-10

**Authors:** Oussama Mouri, Cléa Melenotte, Romain Guéry, Camille Cotteret, Arnaud Schweitzer-Chaput, Alice Perignon, Marc Thellier, Emmanuelle Bourrat, Florentia Kaguelidou, Jean Yves Siriez, Denis Malvy, Jean-Pierre Gangneux, Alexandre Duvignaud, Christophe Ravel, Salvatore Cisternino, Janet Ransom, Eric Caumes, Olivier Lortholary, Max Grogl, Pierre Buffet

**Affiliations:** 1 AP-HP, Hôpital Pitié-Salpêtrière, Service de Parasitologie, Paris, France; 2 Hôpital Necker Enfants Malades, Services de Maladies Infectieuses et Tropicales, Assistance-Publique des Hôpitaux de Paris, Paris, France; 3 Department of Internal Medicine and Infectious Diseases, Hôpital du Confluent, Nantes, Paris, France; 4 Pharmacie, Hôpital Universitaire Necker-Enfants Malades Assistance-Publique des Hôpitaux de Paris, Paris, France; 5 Service des maladies infectieuses et tropicales, groupe hospitalier Pitié-Salpêtrière, AP-HP, Paris, France; 6 Service de dermatologie Hôpital Saint Louis APHP Paris, Paris, France; 7 Service de pédiatre générale Hôpital Robert Debré APHP Paris, Paris, France; 8 Centre d’Investigations Cliniques, INSERM CIC1426, Hôpital Robert Debré, APHP.Nord, Université Paris Cité, Paris, France; 9 Centre d’Investigations Cliniques, INSERM CIC1426, Hôpital Robert Debré, APHP. Nord, Université Paris Cité, Paris, France; 10 Hôpital Robert-Debré, Service d’Accueil des Urgences pédiatriques, Assistance Publique-Hôpitaux de Paris, 48 boulevard Sérurier, Paris, France; 11 Department of Infectious Diseases and Tropical Medicine, CHU Bordeaux, Bordeaux, France; 12 University of Bordeaux, National Institute for Health and Medical Research (INSERM) UMR 1219, Research Institute for Sustainable Development (IRD) EMR 271, Bordeaux Population Health Research Centre, Bordeaux, France; 13 Univ Rennes, CHU Rennes, Inserm, EHESP, IRSET (Institut de recherche en santé, environnement et travail)–UMR_S 1085, Rennes, France; 14 Université de Montpellier, CNRS, IRD, Centre Hospitalo-Universitaire de Montpellier, MiVEGEC, Laboratoire de Parasitologie-Mycologie, CNR Leishmanioses, Montpellier, France; 15 Université de Paris, Necker-Enfants Malades University Hospital, Department of pharmacy, Assistance Publique-Hôpitaux de Paris (AP-HP), Paris, France; Université de Paris, Inserm, UMRS-1144, Faculté de Pharmacie, Optimisation Thérapeutique en Neuropsychopharmacologie, Paris, France; 16 Fast-Track Drugs and Biologics, North Potomac, Maryland, United States of America; 17 Sorbonne Université, INSERM, Institut Pierre Louis d’Epidémiologie et de Santé Publique, Paris, France; 18 Centre de diagnostic, Hôpital de l’Hôtel-Dieu,-Paris, France; 19 Paris University, Necker-Pasteur Center for Infectious Diseases and Tropical Medicine, Necker-Enfants Malades Hospital, AP-HP, IHU Imagine, Paris, France; 20 Unit of Infectious Diseases, Hospital Universitario “12 de Octubre”, Instituto de Investigación Sanitaria Hospital 12 de Octubre (imas12). CIBERINFEC, ISCIII. Department of Medicine, Universidad Complutense, Madrid, Spain; 21 US Naval Medical Research Unit No. 6, Lima, Peru; 22 Centre d’Infectiologie Necker-Pasteur, Institut Pasteur, Paris, France; Uniformed Services University: Uniformed Services University of the Health Sciences, UNITED STATES

## Abstract

**Background:**

In endemic foci, the use of an aquaphilic cream containing paromomycin with/without gentamicin to treat cutaneous leishmaniasis (CL) is safe, painless and cures 78–82% of patients with New and Old World CL. Self-application in travelers requires evaluation.

**Methods:**

Travelers with 1–10 lesions of confirmed CL were prospectively treated with the paromomycin-gentamicin formulation (WR279396, 2012–2017, Group 1) and carefully follow up, or treated with a locally produced paromomycin-only cream (2018–2022, Group 2). The cream was applied once under supervision, then self-applied daily for 20–30 days. A cured lesion was defined as 100% re-epithelialization at day 42 without relapse at three months.

**Results:**

Medical features were similar in Group 1 (17 patients), and Group 2 (23 patients). Patients were infected with either *Leishmania major*, *L*. *infantum*, *L*. *killicki*, *L*. *guyanensis*, *L*. *braziliensis*, or *L*. *naiffi*. Intention-to-treat and per-protocol cure rates were 82% (95% confidence interval (CI) [64.23;100.00]) and 87% (95% CI [71,29;100.00]) in Group 1, and 69% (95% CI [50.76; 88.37]) and 76% (95% CI [57.97; 94.41]) in Group 2. In the pooled Group 1&2, 75% (95% CI [61.58;88.42]) (30/40) and 81% (95% CI [68,46;93.6]) (30/37) of patients were cured in intention-to-treat and per-protocol, respectively. There were no significant differences observed in the success rates between Old World and New World CL (83.3% vs. 60%, p = 0.14). Prospective observations in Group 1 showed that adverse events were mainly pruritus (24%) and pain (18%) on lesions (all mild or moderate). No mucosal involvement was observed in either group.

**Discussion:**

In this representative population of travelers who acquired CL either in the Old or New World, the 81% per-protocol cure rate of a self-applied aminoglycoside cream was similar to that observed in clinical trials.

## Author summary

Self-application of a cream containing antibiotics of the well-known aminoglycoside family (paromomycin and/or gentamicin) cures cutaneous leishmaniasis without injections, but this treatment had not been evaluated yet in travelers. In a group of 40 travelers with cutaneous leishmaniasis we observed that more more than 75% of them cured. Adverse events were mild to moderate pruritus and pain that occurred in less than one fourth of the patients. Our results confirm that widespread availability of a topical aminoglycoside cream would be a simple and effective treatment solution for most adults and children suffering from cutaneous leishmaniasis.

## Introduction

Cutaneous leishmaniasis (CL) have an estimated incidence of 1.5 millions of cases worldwide and is considered a neglected tropical disease affecting the new and old world (OWCL & NWCL) [1,2]. NWCL is commonly caused by the *L*. *viannia* subgenus complex and *L*. *mexicana* complex, while OWCL is commonly due to *L*. *major*, *L*. *tropica* or *L*. *infantum/donovani complex* [3]. Typically, cutaneous leishmaniasis presents as 1 to 10 chronic, infiltrated lesions on exposed parts of the body [1,2]. Wide dissemination is uncommon and visceral spread is exceptional [1,2]. Systemic administration of anti-leishmanial drugs such as meglumine antimonate, pentamidine salts, amphotericin B formulations, or oral miltefosine may result in systemic toxicity. All organs may be involved and serious adverse events include QTc prolongation, hepatitis, increased pancreatic enzymes, and renal toxicities [4–10]. Some cases of death in CL patients were reported related to adverse effects of systemic administration of these anti-leishmanial drugs [4–10]. Systemic treatment has therefore been questioned since cure of CL was observed in 2/3 of travelers managed with local therapy, including cryotherapy in combination with intralesional Sb or paromomycin cream, amongst others [7,9–13].

Local treatment has been recommended by the World Health Organization and the Pan American Health Organization for small lesions, in compliant non-immunocompromised patients who acquired infection outside Bolivia [9,14–20]. Topical aminoglycoside cream was safe and effective in a randomized phase III trial for CL, with a success rate of 75% to 94% (NWCL, 78%, OWCL, 82%) [3,20–23]. Topical paromomycin/aminoglycoside cream is thus an attractive option for treating a mostly benign disease such as CL [1,20–23]. We propose here to evaluate the safety and efficacy of aminoglycoside-based cream in a prospective study and in a real-life cohort of CL travelers, in France.

We conducted successively a prospective, single-arm study to evaluate the efficacy of self-applied WR 279–396 (paromomycin + gentamicin) cream for the treatment of CL in travelers from 2012 to 2016, followed from 2018 to 2021 by analyzing the clinical benefit of a locally produced paromomycin-only cream in the context of routine medical care captured in the National surveillance program of Leishmaniasis (real-life). The main aim was to determine whether self-application of the cream and use of a locally produced formulation was as effective as in previous clinical trials.

## Method

### Ethics statement

For Group 1, the study was sponsored by the Assistance Publique–Hôpitaux de Paris (APHP) with the collaboration of the U.S. Army Medical Research and Materiel Command (USAMMDA) Ft. Detrick in Maryland (Department of the Army, USA). The protocol was approved by the French Human Subjects Review Board “Comité de Protection des Personnes Ile de France VI–Paris” (CPP), by the Agence Nationale de Sécurité du Médicament et des produits de santé (ANSM) and by the Food and Drug Administration (FDA). The study complied with all applicable laws, rules, and regulations of both France and the United States, and by following (or according to) the International Conference on Harmonization Good Clinical Practice Guidelines (ICH-GCP), the Belmont Principles, and the ethical principles that have their origins in the Declaration of Helsinki. The study is registered with ClinicalTrials.gov identifier NCT01988909. Written informed consent was obtained from all study participants and/or guardians before enrollment Patients (or their legal representative) provided verbal or written consent according to national regulations for use of anonymized data on clinical findings, treatment received, clinical outcome and laboratory results. Minors and their legal representatives have also given their consent. For group 2, patients were treated and followed as per national recommendations and data were collected in the context of the Leishmaniasis surveillance program [10]. Patients included in Group 2 respond to the inclusion criteria proposed in the observational study (DR-2013-041; N°912650) approved by the French National Agency regulating data protection (Commission Nationale de l’Informatique et des Liberte´s) [11]. Patients (or their legal representative) provided verbal or written consent according to national regulations for use of anonymized data on clinical findings, treatment received, clinical outcome and laboratory results. No genetic analyses of human DNA were performed [11].

### Study design

This was a 2-step study evaluating the safety and efficacy of topical aquaphilic aminoglycosides cream for the treatment of CL in France. It comprised an initial interventional single arm open-label clinical trial with the WR2789 analyzed in Group 1, followed by an observational prospective cohort of patients receiving a compassionate treatment using a formulation of topical paromomycin produced in France, analyzed in Group 2. Patients were travelers returning to France and attending one of the six participating investigational centers.

*Group 1*: Single arm prospective open-label trial (the study group 2015 to 2016): after establishing eligibility for the study and giving their written informed consent, participants were allocated to receive, once daily, for 20 days the topical cream WR 279,396 (15% paromomycin + 0.5% gentamicin topical cream). Blood tests including hematology, blood chemistries and a serum or urine pregnancy test in all women of child-bearing potential were collected during screening. Blood creatinine levels were measured also at day 1, 7, 14, 20, 28, 42 and 90.

*Group 2*, *observational prospective “real-life” cohort (2018 to 2022)*: From 2018 through 2022, participants were treated once daily, for 20 to 30 days with the locally produced paromomycin formulation. After establishing eligibility to use topical treatment according to the Guidelines, all patient gave their oral informed consent (see Ethic) [9,24]. For patients in Group 2 blood tests were performed at the discretion of the attending physician.

### Study patients

Patients with suspected CL were screened up to 14 days before enrollment for eligibility including parasitological confirmation of ulcerative CL. Eligible patients were non-pregnant or non-breast-feeding men or women, aged 2–80 years, with fewer than 10 lesions, an index lesion, 5 cm >ulcer >1 cm (including induration), confirmed to contain the Leishmania parasite (culture, microscopic examination, or Polymerase Chain Reaction (PCR) of lesion material). The index lesion was designed to evaluate the efficacy of treatment on an ulcer, known to be cause by the parasite *Leishmania*. Subjects with significant preexisting medical conditions, with signs of disseminated cutaneous leishmaniasis, with history of recent treatment (within 8 weeks of starting study treatments) with a recognized anti-leishmanial agent were not included. The index lesion and all other ulcerated lesions were assessed for clinical response by measurement of the length and width of the area of ulceration. A lesion was considered as completely cured if 100% re-epithelialization was observed. Non-ulcerated lesions were also measured to monitor the total area of exposure of lesions to study drug and were evaluated for cure (regression of induration) in percentage.

### Parasitological diagnosis and parasite species identification

Samples were obtained from lesions by scraping and aspiration. Specimens were smeared onto microscope slides or placed in a culture medium, and both were analyzed at the parasitology laboratory. Evidence of infection with *Leishmania* was documented by either microscopic identification of *Leishmania* amastigotes on Giemsa-stained slides, or by the demonstration of motile *Leishmania* promastigotes in culture, or by amplification of parasite DNA by PCR as described. Briefly, real-time polymerase chain reaction (RT-PCR) was performed on a *Leishmania* kinetoplast DNA target using the following primers and probe: 15 pmol of direct primer (CTTTTCTGGTCCTCCGGGTAGG), 15 pmol of reverse primer (CCACCCGGCCCTATTTTACACCAA), and 50 pmol of TaqMan probe (FAM-TTTTC GCAGAACGCCCCTACCCGC-TAMRA) [25]. Whenever possible, Leishmania species identification in culture samples was performed by mass spectrometry, restriction fragment length polymorphism (RFLP) or Multi Locus Sequence Typing (MLST) as described [26–28].

### Drug administration

WR 279,396 (15% paromomycin + 0.5% gentamicin topical cream) was manufactured by Teva Pharmaceuticals USA, Sellersville, PA, in accordance with Good Manufacturing Practice [29]. Tubes were kept between 2 to 6°C before use. The local French formulation of topical paromomycin was produced by the Necker Hospital Pharmacy department, according to French Good Preparation Practice. Topical paromomycin formulation is adapted from the formulation tested in large clinical trials [21]. Cream jars were kept in the patient refrigerators before use [21]. The first application of the cream was supervised at the hospital enabling tutoring patients on the application process. All other applications were performed at home by the patient or by relatives for both groups; all lesions (*i*.*e*., the index lesion plus any non-index lesion) were treated topically once daily for 20 days and for 20–30 days for group 1 and 2 respectively. Before the application of the cream, the lesions were cleaned with soap and water, debrided if necessary, then dried. The cream was applied on each lesion to cover the ulcerated area and the surrounding indurated border before applying the dressing as previously reported [3]. For both groups, 1 and 2, a cheap, adhesive occlusive dressing (Neoplaste, ADELS, Sousse, Tunisia, or Urgotul Lite, Urgo, Paris, France) was applied to protect lesions and clothes and prevent the cream from being wiped out. For group 1, the cream tubes were weighed before and at the end of treatment and the result was recorded in the case report form (group 1). For Group 2, patients disposed of cream jars. Topical paromomycin formulation is described at the S1 Material and Methods.

### Follow-up and toxicity evaluation

Upon screening, all patients in Group 1 had an evaluation of blood parameters including ALT, AST, creatinine, potassium, sodium, glucose, hemoglobin, platelets and white blood cell counts upon screening. Blood creatinine was repeated on day 20 as aminoglycosides have the potential for renal toxicity. Patients in Group 1 consulted once-a-week at the hospital during the treatment period (20 days), one week after the end of treatment (day 28) then at day 42 and day 100 to assess toxicity and measure the size of lesions. Thus, kinetic endpoints of the clinical response were proposed for patients included in Group 1. Patients in Groups 2 were followed by their attending physician [10]. Efficacy was assessed by measuring the CL lesion area (calculated according to the following formula: index lesion area (mm2) = length (mm) x width (mm) where length and width are the largest and the smallest diameters of the induration at baseline, at the end of the treatment period on day 20, and at follow-up visits on days 28, 42 and 100 (end of study)). Application sites (CL lesions and surrounding areas) were inspected upon each visit. The presence and intensity of erythema, edema/swelling, and any other clinical sign were recorded. Patients in Group 1 were asked to grade their pain using the Wong-Baker FACES pain rating scale. If medications had been used to treat one or several side effects, these were recorded. Serum creatinine was measured at day 20 to assess potential aminoglycoside-related renal toxicity. In Group 2, the evaluation of safety was made by the attending physician [10].

### Study endpoints

Complete clinical cure was defined as either 100% re-epithelialization (i.e., a 0 x 0 length x width measurement) of the index lesion on day 42 (±7) visit or initial clinical improvement (*i*.*e*., >50% re-epithelialization) by day 42 followed by complete re-epithelialization on day 100 (±14) at the latest, and no further relapse at the end of the trial (100 days). Relapse was defined as a 10% or greater increase in the area of ulceration or a shift from 100% to < 100% re-epithelialization of the index lesion at any time between day 42 and day 100. The primary efficacy endpoint, evaluated weekly, was the proportion of participants achieving complete clinical cure in both groups. Any other situation was defined as a treatment failure. If the patient was withdrawn from the study, he/she was considered as a treatment failure in the intention-to-treat analysis. If a new lesion occurred during the treatment period, local therapy was applied on that lesion as well. Secondary endpoints included: the proportion of participants in whom all treated lesions met the definition of complete final clinical cure; the proportion of all treated lesions met the definition of complete final clinical cure (independently of unique patients). The safety endpoints were adverse events in general, local reactions at the treated sites, and aminoglycoside renal toxicity determined by serum creatinine measurements at the end of therapy on day 20.

### Statistical analysis

Descriptive statistics were expressed as percentages for categorical variables and as mean with standard deviation and median with interquartile range for continuous variables. Demographic variables, underlying conditions and clinical characteristics were compared using of the Pearson’s Chi square test or Fisher’s exact test for categorical variables or the T-test for continuous variables. Results with p values < 0.05 were considered statistically significant. The intention-to-treat/safety datasets included all patients who received any administration of paromomycin cream (group 1 and 2). The per-protocol dataset included all patients who received daily doses of paromomycin cream (group 1 and 2) for at least 18 of the total 20 days and had lesion measurements at day 42 and 100, and who did not receive rescue medications to treat leishmaniasis at any time during the study. As these were open label single group studies, there were no statistical hypotheses or comparisons of efficacy outcomes. GraphPad PRIMS Software Version 7.0a was used for Figs.

## Results

### Characteristics of patients

Group 1 received 279,396 WR and included 17 patients, whereas group 2 included 23 patients involved in the paromomycin cream treatment program of the French leishmaniasis network. Demographic and clinical characteristics of patients were compared during two different periods, 2015–2016 for Group 1 and 2018–2021 for Group 2 (**Table 1)**. No significant difference was observed for any of the variables, including the continent of infection, age, the proportion of children, gender, the prevalence of preexisting conditions, the number of lesions and the size (Table 1).

**Table 1:** Baseline demographics and main clinical features.

	Group 1 Prospective study with WR279396	Group 2 Treatment program with local formulation of paromomycin cream
	2015–2016	2018–2021
N	17	23
N (%) female	7/10 (41)	8/23 (35)
Median age (range)	36 (3–68)	29 (5–87)
Mean age (±SD)	35.38 ± 22	36.2±23.7
N (%) children (< 15 yo)	3 [18]	4 [17]
Mean lesion number (N = 164)	2.8 [1]	2.4
Median lesion number (N = 164)	2	2
N (%) patients with > 10 lesions (N = 164)	0	0
Mean lesion number if >10 excluded (N = 157)	2.8	2.3
Median lesion duration (months) (N = 82)	3.5	3.5
N (%) ulcerative lesions (N = 167)	17 (100) [2]	17 (73)
Median ulceration area (mm2)(N = 132)	265	165
N (%) patients infected in the New World (N = 166)	6 (35)	8 [34]
• No significant difference (NS) (p-value = 0.32, 0.84, 0.34 respectively) • As per protocol, all index lesions had to be ulcerated but in patients with multiple lesions, some lesions were not ulcerative, leading to a total % of ulcerative lesions of 38/47 (81%)		

**Fig 1:**
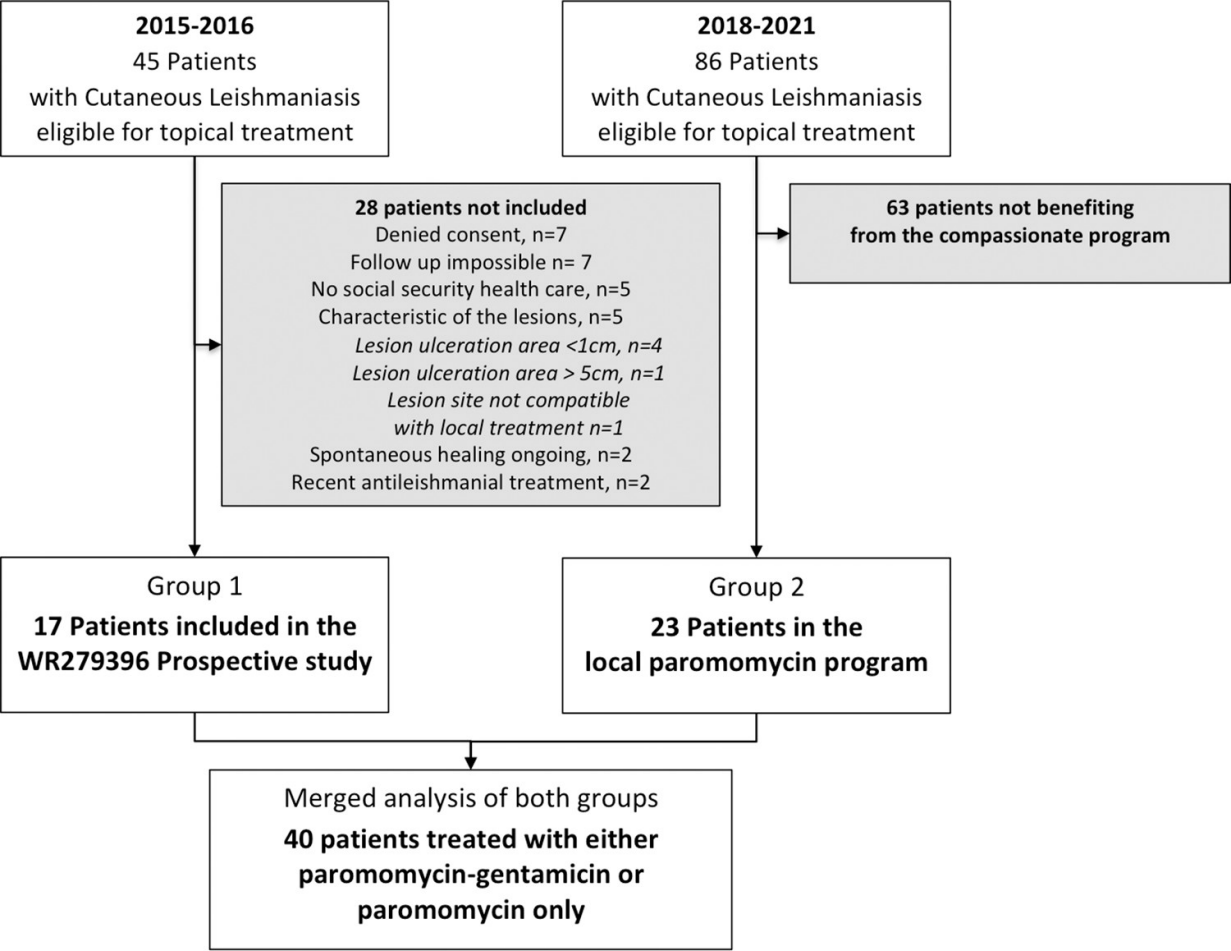
Study flowchart for Group 1 & 2.

**For the group 1,** 17 among the 45 patients screened were included and allocated to receive once-daily topical applications of WR 279,396 (15% paromomycin + 0.5% gentamicin) for 20 days (**Fig 1**). Ten patients were male (58.8%) and 7 were female (41.2%). Mean age was 35,38 ± 22 years old and 3 children were included with a median aged of 3 year-old (range 3–5 years). In total, the 17 patients had 47 lesions, 81% of which were primarily ulcerative. The mean number of lesions per individual was 2.8 (range 1 to 9). The mean (standard deviation–SD) ulcer area of the index lesion was 265.4 mm^2^ (327.1 mm^2^). Index lesions were predominantly on the arms (35.3%), legs (23.5%), or face (17.6%). Lesions were first identified 108 days (range 26 to 306 days) prior to the start of study treatment (**Table 1**). The infecting *Leishmania* species were identified in 16 of 17 (94%) enrolled patients; 11 (65%) and 6 (35%) were acquired in the Old and New World respectively. *L*. *major* was the most frequently identified species *(7 isolates)*, followed by *L*. *infantum* [2], and *L*. *killicki* [1], and *L*. *guyanensis/panamensis* [3], and *L*. *braziliensis/peruviana* [2], and L. *naiffi* [1]. One isolate was identified as *Leishmania sp*. *Old World complex* (S1 Table**).**

**In Group 2,** among the 23 patients included, 16 were men, mean age was 36.2±23.7 year-old. Three were children (5, 6 and 8 years-old) and the mean number of lesions per patient was 2,3. The median duration of lesions was 105 days. Eight patients had NWCL and 15 OWCL. None of these patients had mucosal involvement at the time of inclusion and during follow-up. The infecting *Leishmania* species were identified for 16 participants. Here *L*. *infantum* was the most frequently identified species (5 isolates) followed by *L*. *major* [4], *L*. *guyanensis* [2], *L*. *viannia* subgenus [2], *L*. *braziliensis* [1] *L*. *mexicana* [1], *L*. *panamensis* [1] while 7 were undetermined.

### Efficacy

**Among the 40 patients analyzed, when Groups 1 & 2 were pooled**, 75% (95% CI [61.58;88.42]), (30/40), and 81% (95% CI [68,46;93.6]), (30/37), of patients were cured in the intention-to-treat and the per-protocol, respectively.

**Fig 2:**
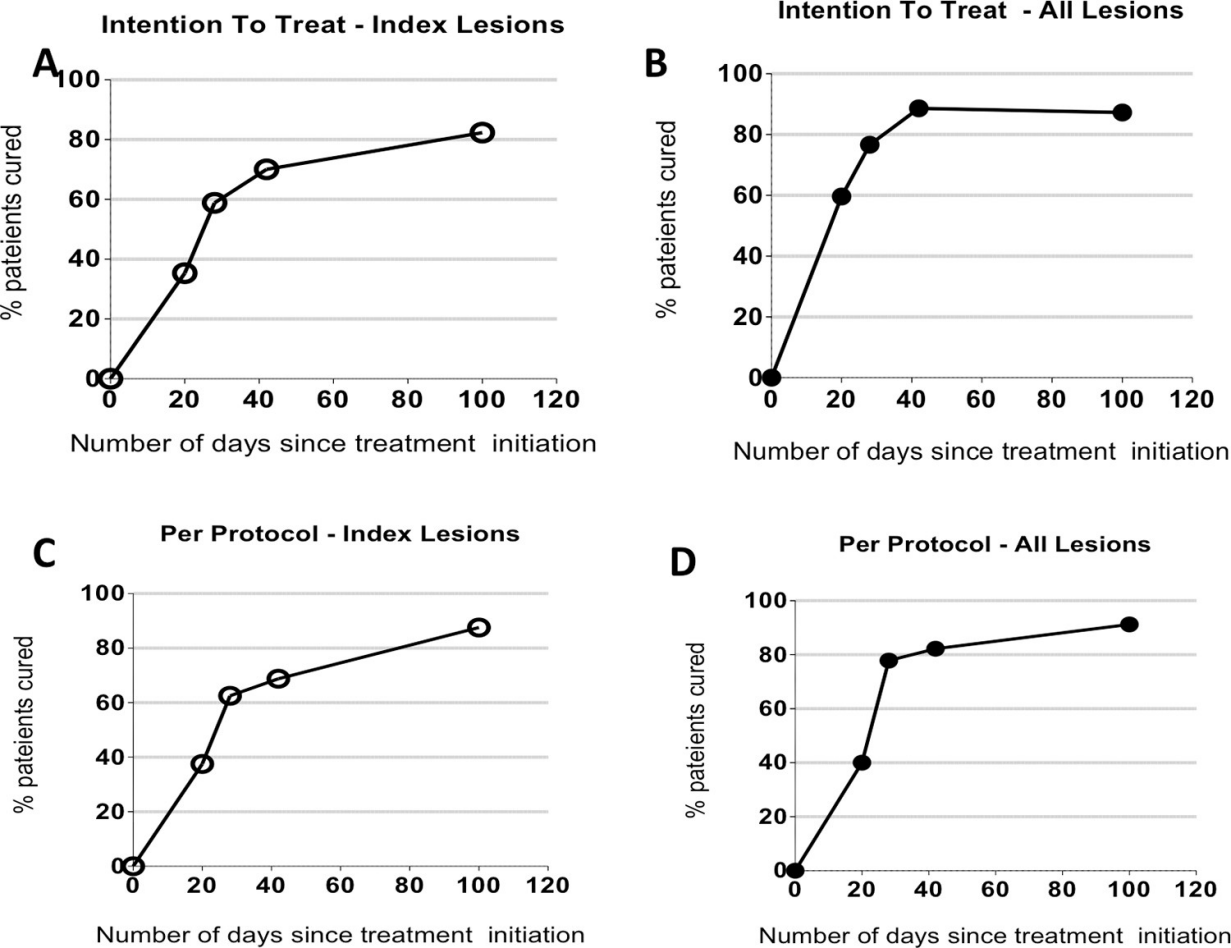
**Lesions cure rate over time (Panel A. B). Lesions Cure Rate over Time (Intention-to-treat**). Percentage cure of both index and all lesions that met criteria for clinical cure at each scheduled follow-up visit during the study. Clinical cure (intention-to-treat analysis) of index lesions occurred as early as day 20, the last day of drug application, at a rate of 35.3% for the ITT dataset. By day 42, 70% of index lesions were cured, while 88.6% of all lesions met clinical cure criteria. At day 100, the clinical cure rate for index lesions and all lesions reached 82.3% and 87.2% in ITT patients, respectively. **(Panel C.D) Lesions Cure Rate over Time (Per Protocol**). Percentage cure of both index and all lesions that met criteria for clinical cure at each scheduled follow-up visit during the study. Clinical cure (Per Protocol analysis) of index lesions occurred as early as day 20, the last day of drug application, at a rate of 37.5% for the Per Protocol dataset. By day 42, 68.75% of index lesions were cured, while 82.2% of all lesions met clinical cure criteria. At day 100, the clinical cure rate for index lesions and all lesions reached 87.2% and 91.2% in Per Protocol patients, respectively.

In Group 1, 14 of 17 (82.3%, 95% CI [64.23;100.00]) index lesions met the criteria for the complete clinical cure in the intention-to-treat analysis **(**S2 Table**)**. All these 14 patients also had a complete clinical cure of all lesions. Complete clinical cure in the per-protocol analysis was 14 of 16 (87.5% 95 CI [71,29;100.00]) index lesions, which were again the same rate as when considering all lesions. When evaluating the complete clinical cure of all lesions by-lesion instead of by-patient, a total of 41 of 47 (87.2%95 CI [77,69; 96,77]) lesions in the ITT and 41 of 45 (91.2% 95 CI [82,8;99.43]) in the per-protocol analysis met the criteria **(Fig 2)**. Three patients did not meet the primary efficacy endpoint, one had an unclosed index lesion, one required additional intralesional therapy, one required intramuscular treatment (S1 Results). No mucosal involvement was observed in this patient during treatment and upon final follow-up 30 months later.

In Group 2, 16 among the 21 patients included were considered as cured, *i*.*e*. 76% (95% CI [57.97; 94.41]) in the per-protocol analysis (available information for 21 of the 23 patients) and 16/23, 69% (95% CI [50.76; 88.37]), in the intention-to-treat analysis. Five patients had a negative clinical outcome. One of them relapsed and required cryotherapy, one required additional treatment with intralesional meglumine antimoniate, one required muscle/systemic meglumine antimoniate injection, and information was not available for two patients. None of these patients experienced any adverse events during or after treatment. All physicians were contacted on the phone 24–36 months after treatment. Seven responded and no relapse was identified at the last visit, after 36 months of follow-up for 2, 24 months for 1, 12 months for 2, 6 and 3 months for 1 patient respectively. No patient had signs or symptoms of mucosal involvement, and all had inactive scars.

### Kinetics of cure Group 1

**Fig 3:**
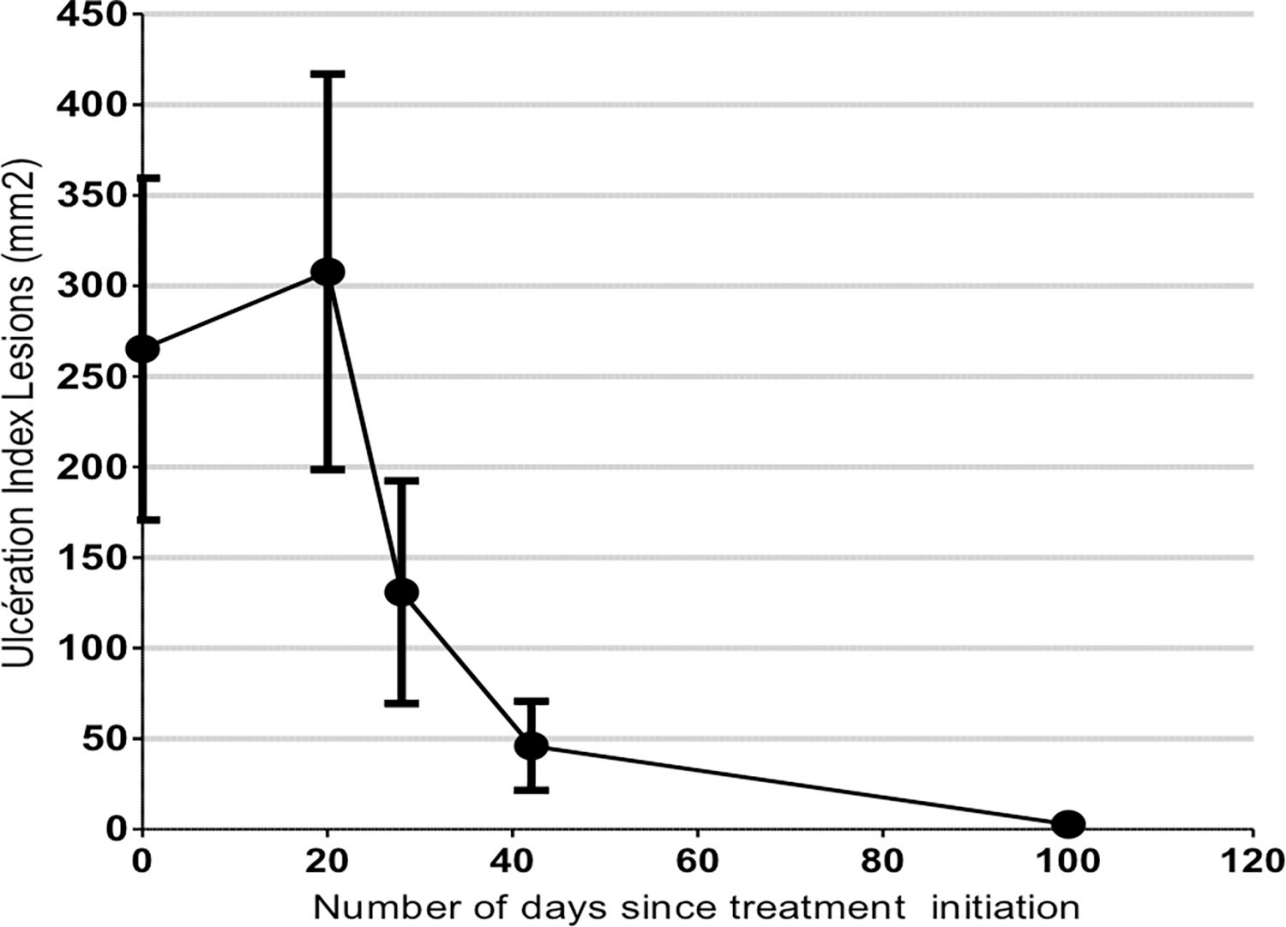
Evolution of the index lesion over time during the study. In response to the drug treatment, lesion area increased for the index lesion during the treatment period (20 day), then decreased after the cream application period ended. By day 28, the mean index lesion size reduced by approximately 40% compared with the baseline lesion size. By day 28, the mean index lesion size reduced by approximately 50% and 99% by day 100 compared with the baseline lesion.

**Fig 4:**
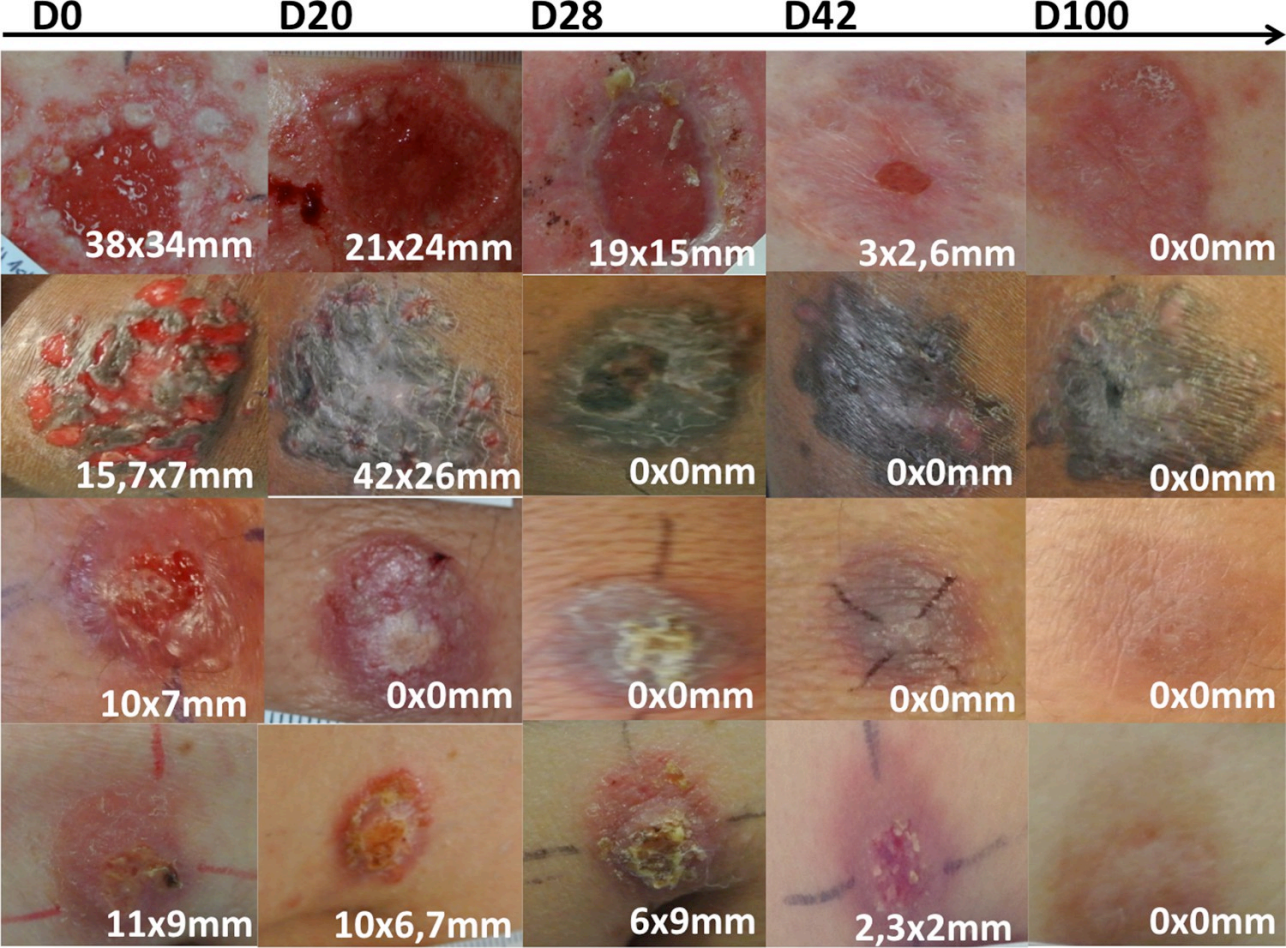
Summary in pictures of the evolution of the index lesion of patients treated with cream over the time during the study. The squares in green indicates patients who meet the criterion for final clinical cure of the index lesion.

Among the 17 patients included (Group 1), kinetics of lesion progression was performed. Interestingly, at the end of the treatment (day 21) only 6/16, 37.5% (95%CI [13,78–61,22]) of the lesions were healed for the per protocol dataset, and 11/16, 68.75% (95% CI [46,04–91,46]) of index lesions were healed at Day 42, while 13/16, 81.2% (95% CI [62,12–100]) of all lesions met clinical cure criteria at the same time. At day 100, the clinical cure rate for index lesions and all lesions reached and 15/16, 93.75% (95% CI [81,89–100]) and 15/17, 88.2% (95% CI [64,24–100]) per-protocole and in intention-to-treat analysis, respectively (Fig 2). Regarding the size of the index lesion, the mean index lesion size reduced by approximately 50% by day 28 and 99% by day 100 compared with baseline (Figs 3 and 4). Thus, complete clinical cure was observed between day 42 and day 100, *i*.*e*. 2 to 11 weeks after the last application of the cream (S1 Results).

### Efficacy in New World *versus* Old World CL

For 13 of 17 patients included in Group 1 and 21 of the 23 patients in Group 2, information on both clinical cure and geographical origin (New and Old World) was available. Among Group 1, 10 patients (77%) were cured overall, including 2 of the 4 and 8 of the 9 who had been infected in the New and Old World, respectively. In Group 2, 16 patients (76%) were cured, including 4 of the 6 and 12 of the 15 who had been infected in the New and Old World, respectively. Upon a pooled analysis of Groups 1 & 2 (34 evaluable patients), cure rates were 6/10 (60%, CI 95% [28,38–91,62]) and 20/24 (83.3%; CI 95% [68,42–99,24]) for infections acquired in the New and Old World, respectively (p = 0.14; Chi2 test).

### Development of new lesions

In group 1, two patients developed one new lesion each. One patient had acquired CL in South America (*L*. *guyanensis*). A new lesion appeared on day 61 was treated topically with WR279,396 once daily for 20 days and cured on day 88. The second patient acquired CL in Tunisia (*L*. *infantum*). The new lesion appeared on day 16 and was treated topically with WR279,396 once daily for 20 then cured on Day 28 (S2 Table). No new lesion was observed in Group 2 patients, during the follow up.

### Safety

Prospective observations in Group 1 showed a total of 9/17 patients (52.9%, 95 IC [29,21–76,67]) were reported to have at least one adverse event (AE). AEs were mainly pruritus (24%) and pain (18%) on lesions. A total of 8 (40,06% 95% IC [23,33–70,79]) patients had mild AEs and 5 (29.4% 95IC [7,75–51,7]) had moderate AEs. Two patients (12%) had serious AEs (S4 Table). One developed a pregnancy during treatment. One other presented a rash attributed to chigger bites (S1 Adverse Events). A total of 31 AEs were reported in Group 1. All were mild (54.8%, 95% IC [37,32–72,2–36]) or moderate (45.2%, 95% IC [27,64–62,68]) in severity (S3 Table). There were no death and no severe or life-threatening AEs reported in either group. No patients were withdrawn due to AEs.

## Discussion

We report here a cohort of patients with CL (with one or multiple lesions) treated with an aminoglycoside-based cream as a single topical treatment. Just less than half of patients were women, a sixth were children, all patients had a median of 2 lesions and a third of them were infected in the NW, which is quite representative of the French travelling population [10,11]. In this group representative of the general population of patients with imported CL, infected with a wide diversity of Leishmania species *(L*. *major*, *L*. *infantum*, *L*. *killicki*, *L*. *guyanensis*, *L*. *braziliensis*, *or L*. *naiffi)*, topical treatment with aminoglycosides was simple, safe and effective. We observed that 75% of patients were cured in the intention-to-treat analysis. This observation is consistent with previous reports that sustainably showed cure rates ranging from 74 to 87%, since 1994 and including large placebo-controlled studies [1,3,10,21,22]. From a pragmatic point of view, 70% of patients did not require systemic anti-leishmanial treatment to achieve sustained cure, which corresponds to a significant simplification of patient management. The cure rate reached with topical paromomycin cream is similar to that observed with local therapy, 70 to 79% [10,28,30].

The safety of aminoglycoside-based creams, captured in detail in Group 1, was very good. The types and frequencies of AEs observed, pruritus and pain on lesions (always mild or moderate), were similar to those in prior clinical trials, affecting less than 25% of patients [1,21]. Only two serious events unrelated to the cream application were reported: a generalized rash due to chigger bites and a pregnancy starting during drug application, which went to full-term uneventfully. Although new world patients are more often treated with systemic treatment due to fear of mucosal leishmaniasis as a late complication, this did not occur in any of our patients. Indeed, no mucosal involvement was observed in this cohort of 40 patients, while 14 of them were infected with either *L*. *braziliensis*, *L*. *panamensis/guyanensis*, or *L*. *infantum* and followed for 3 months to 3 years.

In addition of being effective and safe, self-applied aminoside-based creams are time- and resource-saving for patients and health-care providers. Self-application limits indeed the number of health-care interventions for treatment, reducing care-related costs. As opposed to most current options for the local therapy of cutaneous leishmaniasis, applying an ointment does not require the use of needles or expensive devices. A painless option is especially advantageous in children, who account for the majority of patients in endemic areas. Not least, patients with up to 10 lesions can be treated, *i*.*e*., more than the 4 lesion-limit of most recommendation for local therapy. Taken together, these tangible advantages markedly widen the target population who can benefit from a prompt, painless and effective treatment and enhance the public-health benefit of programs.

Patients should be informed that the area of ulceration may increase during treatment (likely related to the mildly irritative effect of the formulation rather than to a rarely observed full-blown eczema), but that healing will generally occur in a few days/weeks after the end of applications, that should therefore be maintained with compliance during the 3–4 weeks recommended treatment period. There was a non-significant difference in treatment response between OWCL and NWCL (83% versus 60%, respectively). This observation is not entirely consistent with previous reports in the NW. In Panama, the cure rate of NWCL caused by *L*. *panamensis* and treated with paromomycin-gentamycin, was 79% [1,3,31]. In Ecuador, paromomycin plus methylbenzethonium chloride cream induced cure in 79 to 85% of CL patients infected with *L*. *panamensis*, *L*. *guyanensis*, or *L*. *braziliensis* [31–34].

This work has some limitations. This study had an unusual structure, with Group 1 and Group 2 being treated with different cream formulations for different periods of time, with no direct comparison to a positive or negative control group. A placebo-controlled group was no longer ethically acceptable, as several Phase 2 and pivotal Phase 3 studies have now undisputedly shown the efficacy of aminoglycoside creams both in OWCL and NWCL. A positive control was very difficult to determine as no currently available treatment option in CL comes close to aminoglycoside-based cream in terms of risk-benefit ratio. Therefore, an open design was the most reasonable way to gather information on the performance of a simplified, less supervised application approach. In this study, Group 1 was a single-arm prospective open-label trial analysis and Group 2 was a prospective "real-life" cohort analysis. This generated quality differences between the two groups analyzed. However, merging these 2 experiences in a single report clearly illustrates that a progressive shift in treatment procedures from the controlled applications in a clinical trial to real-life applications by outpatients in (almost) current practice did not markedly alter treatment efficacy, assessed in both groups using the same outcome definition. This reports also shows how a team copes with elusive drug access in neglected diseases. Aminoglycoside-based creams are by far the most user-friendly option for the local treatment of CL, and benefit from the most robust evaluation by appropriately designed and executed comparative, prospective trials [3,22,29]. Yet, more than 10 years after the publication of the first pivotal Phase 3 trial a widely available formulation still does not exist [21].

Another limitation was the relatively small number of patients limited the power of this study. The difference in cure rates observed between NWCL and OWCL may therefore have been statistically insignificant either because of a lack of difference or a lack of statistical power. Further studies in the specific context of imported CL, with careful control of confounding parameters such as the parasite strain, host factors and the composition of the topical product are needed to address this issue. Finally, the inavailability of a standardized, industrial formulation was an unsolvable issue, leading to the use of a locally produced cream during the second phase of the study. On the other hand, using a non-industrialized formulation of aminoglycoside-based cream implies a potential lack of standardization in the composition of the product. The industrial partner now involved in the production of a paromomycin cream has not yet delivered a widely accessible formulation. This last point is crucial because it underlines the need of a standardized and homogeneous industrialized paromomycin cream to safety treat CL, regardless of the geographical location. To conclude, our results confirm that wide availability of aminoglycoside-based topical treatment of CL would positively impact a vast population of patients, especially children, who would benefit from a prompt, painless and effective treatment with robust public-health benefit, either directly by reducing symptoms and stigma, and indirectly in *L*. *tropica* foci, by potentially reducing the reservoir of parasite carriers.

## Supporting information

S1 Material and Methods Topical paromomycin formulation Group 1 and Topical paromomycin formulation Group 2. (DOCX)

S1 Results Efficiacy. (DOCX)

S1 Adverse Events Patient 1: A 20-year-old woman had a single CL lesion with no significant medical history. (DOCX)

S1 Table Infecting Leishmania species (Group 1). (DOCX)

S2 Table Final Clinical Cure of Index Lesions and All Lesions (Group 1). (DOCX)

S3 Table Number of Adverse Events. (DOCX)

S4 Table Listing of Serious Adverse Events. (DOCX)
