# Supplementary material for: Self-application of aminoglycoside-based creams to treat cutaneous leishmaniasis in travelers
Source: PLoS Negl Trop Dis. 2023 Aug 10;17(8):e0011492. doi: 10.1371/journal.pntd.0011492 (PMC10443860; doi:10.1371/journal.pntd.0011492)
Supplement: S1 Material and Methods — (DOCX) [file pntd.0011492.s001.docx]

**Supplemental S1_Material and Methods**

**Topical paromomycin formulation Group 1**The formulation of the cream used for group 1 is explicit below and uses as a base the composition of the widely available cream “Aquaphilic”.

Below is further manufacturing description of the optimal paromomycin/gentamicin topical

formulation.

Formulation 232 contains:

Paromomycin Sulfate 15.00%'

Aquaphilic with

10% Carbamide Ointment2 67.80%

Water 16.67%

Footnotes:

1 All percent compositions are weight percent

2 Available from Medco Lab., Inc., Sioux City, Iowa 51103

Aquaphilic with 10% Carbamide Ointment has the following composition:

Sorbitol - 4% Isopropyl Palmitate - 0.5%

Propylene Glycol - 6% Stearyl Alcohol - 19%

Water - 39.85% White Petrolatum - 19%

Urea - 10% Propyl Paraben - 0.15%

Lactic Acid - 0.5% Methyl Paraben - 0.25%

Sodium Lauryl Sulfate - 0.75%

Method of Preparation for 150 g of Formulation 232:

1. Weigh 22.5 g of paromomycin sulfate and 0.75 g of gentamicin sulfate.

2. Weigh 101.7 g of Aquaphilic with 10% Carbamide Ointment.

3. Heat 25 ml of distilled water to about 70'C.

4. Heat Aquaphilic with 10% Carbamide Ointment in a separate container to about 70'C.

5. Dissolve paromomycin sulfate and gentamicin sulfate in the heated water with stirring (about

0.5 - 1 min.) and remove solution from heat.

6. Remove Aquaphilic with 10% Carbamide Ointment from the heat and add the aqueous

solution of paromomycin sulfate and gentamicin sulfate with stirring. Stir for 10 minutes with

an overhead stirrer to incorporate the aqueous solution into the base as it cools.

**S1_Topical paromomycin formulation Group 2**

We used the same formulation except that Gentamicin was not added because its presence in the cream does not influence efficacy, as shown in Ben Salah et al. NEJM 2013 [1]

Paromomycin Sulfate 15%

Aquaphilic with

10% Carbamide Ointment (available from Medco Lab., Inc., Sioux City, Iowa 51103 ),

10% Carbamide Ointment has the following composition:

Sorbitol – 4%

Propylene Glycol - 6%

Water - 39.85%

Urea - 10%

Lactic Acid - 0.5%

Sodium Lauryl Sulfate - 0.75%,

Isopropyl Palmitate - 0.5%

Stearyl Alcohol - 19%

White Petrolatum - 19%

Propyl Paraben - 0.15%,

Methyl Paraben - 0.25%.
