# Supplementary material for: Self-application of aminoglycoside-based creams to treat cutaneous leishmaniasis in travelers
Source: PLoS Negl Trop Dis. 2023 Aug 10;17(8):e0011492. doi: 10.1371/journal.pntd.0011492 (PMC10443860; doi:10.1371/journal.pntd.0011492)
Supplement: S1 Results — (DOCX) [file pntd.0011492.s002.docx]

**S1 Results**

S2_Efficacy **In group 1,** 3 patients did not meet the primary efficacy endpoint. In the first patient with a *L. braziliensis* leg lesion, treatment was considered unsuccessful because the index lesion was not completely closed at Day 100. The patient refused to receive another treatment and was cured at Day 115 without further relapse or mucosal involvement upon final follow-up 36 months later. The second patient had a lesion of the wrist that was re-epithelialized on Day 42 but with new active peripheral papules containing parasites (*L. major*). Healing was achieved with superficial cryotherapy plus intra-lesional injections of meglumine antimoniate, as per current French recommendations. The third patient had 3 large ulcers of the lower limbs due to *L. guyanensis.* On Day 28, ulcerations had increased in size. Healing was achieved with intramuscular isethionate (4mg/kg every other day for 5 days, i.e., 3 injections[2]).

**In Group 2,** five patients had negative clinical outcome.

One presented relapse of the ulcerative lesion at the end of the treatment, and finally cured with cryo-therapy. A second patient had partial improvement (>75% at 3 months) and treatment was completed with one intra-lesion injection of glucantime. A third patient was ultimately cured with intra-muscular/systemic injection of glucantime. Information was not available for two patients. None of these patients experienced any adverse events during or after treatment. All physicians were contacted on the phone 24 – 36 months after treatment. Seven responded and no relapse was identified at the last visit, after 36 months of follow-up for 2, 24 months for 1, 12 months for 2, 6 and 3 months for 1 patient respectively.

**Supplemental informations about the kinetics of the lesions**

For these 17 patients, the median lesion area increased by 16% during the treatment period (Day 20 compared with Day 1) for index lesions. This was rapidly followed by a decrease in the ulceration area just after the end of cream applications. By day 28, the mean index lesion area was reduced by 51% compared to the baseline lesion size (Figure 3). In patients who acquired their lesion in the NW; the lesion area increased during the treatment period (Day 20 compared with Day 1) for index lesions. This was also rapidly followed by a decrease in the ulceration area just after the cream applications ended. In patients with Old World CL index lesions area started to decrease during the treatment period and continued to decrease afterward. Four of the 5 patients infected in the NW could be contacted on the phone 24 – 36 months after treatment. None had signs or symptoms of mucosal involvement, and all had inactive scars.

S2_Adverses events
Patient 1 : A 20-year-old woman had a single CL lesion with no significant medical history. During the screening, she had a negative pregnancy test and was using a hormonal contraceptive (ethinyl estradiol and levonorgestrel), that she stopped 9 days before the end of the topical cream application (study day 11) without informing the investigator. On day 19, she had a positive pregnancy test. The starting date of the pregnancy was estimated to be day 15 of drug application. She had a normal delivery 8 months and a half after conception with no evidence of birth defects. The investigator-assessed pregnancy as not related to the investigational cream. The CL lesion cured by Study day 28 and the patient completed the full follow-up.

Patient 2: A 68 year-old male had a single CL lesion and no other significant medical history. He received 20 daily applications of the topical cream, on day 29 he had a papulo-nodular rash without pruritus that resolved on day 40 without any treatment. The rash was attributed to many chigger bites while on a hunting trip. The investigator-assessed the rash as not related to the investigational cream.
