# Supplementary material for: Self-application of aminoglycoside-based creams to treat cutaneous leishmaniasis in travelers
Source: PLoS Negl Trop Dis. 2023 Aug 10;17(8):e0011492. doi: 10.1371/journal.pntd.0011492 (PMC10443860; doi:10.1371/journal.pntd.0011492)
Supplement: S1 Adverse Events — (DOCX) [file pntd.0011492.s003.docx]

S1 Adverses Events
Patient 1 : A 20-year-old woman had a single CL lesion with no significant medical history. During the screening, she had a negative pregnancy test and was using a hormonal contraceptive (ethinyl estradiol and levonorgestrel), that she stopped 9 days before the end of the topical cream application (study day 11) without informing the investigator. On day 19, she had a positive pregnancy test. The starting date of the pregnancy was estimated to be day 15 of drug application. She had a normal delivery 8 months and a half after conception with no evidence of birth defects. The investigator-assessed pregnancy as not related to the investigational cream. The CL lesion cured by Study day 28 and the patient completed the full follow-up.

Patient 2: A 68 year-old male had a single CL lesion and no other significant medical history. He received 20 daily applications of the topical cream, on day 29 he had a papulo-nodular rash without pruritus that resolved on day 40 without any treatment. The rash was attributed to many chigger bites while on a hunting trip. The investigator-assessed the rash as not related to the investigational cream.
