# Supplementary material for: Self-application of aminoglycoside-based creams to treat cutaneous leishmaniasis in travelers
Source: PLoS Negl Trop Dis. 2023 Aug 10;17(8):e0011492. doi: 10.1371/journal.pntd.0011492 (PMC10443860; doi:10.1371/journal.pntd.0011492)
Supplement: S1 Table — (DOCX) [file pntd.0011492.s004.docx]

**S1 Table. Infecting Leishmania species (Group 1)**

| *Leishmania* Species | N (%) | |
| --- | --- | --- |
|  | **ITT Patients N = 17** | **PP Patients N = 16** |
| *L major* | 7 (41.2) | 7 (43.75) |
| *L killicki*  *L infantum* | 1 (5.9)  2 (11.8) | 1 (6.3)  2 (12.5) |
| *L guyanensis*  *L braziliensis*  *L naiffi* | 3 (17.6)  2(11.8)  1 (5.9) | 2 (12.5)  1 (6.3)  1 (6.3) |
| *L.* *sp* | 1 (5.9) | 1 (6.3) |

*Leishmania* species identification in culture samples was performed by mass spectrometry or by restriction fragment length polymorphism MLST PCR as described [3,4]
