# Supplementary material for: Self-application of aminoglycoside-based creams to treat cutaneous leishmaniasis in travelers
Source: PLoS Negl Trop Dis. 2023 Aug 10;17(8):e0011492. doi: 10.1371/journal.pntd.0011492 (PMC10443860; doi:10.1371/journal.pntd.0011492)
Supplement: S2 Table — (DOCX) [file pntd.0011492.s005.docx]

S2 Table Final Clinical Cure of Index Lesions and All Lesion (Group 1)

|  | Intention-to-treat | Per-protocol |
| --- | --- | --- |
| Patients Meeting Criteria for Final Clinical Cure, n (%)  N=17 N=16 | | |
| Index Lesion | 14 (82.3) | 14 (87.5) |
| All Lesions | 14 (82.3) | 14 (87.5) |
| Lesions Meeting Criteria for Final Clinical Cure, n (%) | | |
|  | N=47 | N=45 |
| **All Lesions** | 41 (87.2) | 41 (91.1) |
