# Supplementary material for: Self-application of aminoglycoside-based creams to treat cutaneous leishmaniasis in travelers
Source: PLoS Negl Trop Dis. 2023 Aug 10;17(8):e0011492. doi: 10.1371/journal.pntd.0011492 (PMC10443860; doi:10.1371/journal.pntd.0011492)
Supplement: S3 Table — (DOCX) [file pntd.0011492.s006.docx]

S3 Table Number of Adverse Events

|  | | Total number (%) |
| --- | --- | --- |
| **Total AEs** | | 31 (100) |
|  | Mild AEs | 17 (54.8) |
|  | Moderate AEs | 14 (45.2) |
|  | Severe AEs | 0 (0) |
|  | Life-threatening AEs | 0 (0) |
| **Patients with any AE** | | 9 (52.9) |
|  | Patients with mild AEs | 8 (47.1) |
|  | Patients with moderate AEs | 5 (29.4) |
|  | Patients with severe AEs | 0 (0) |
|  | Patients with life-threatening AEs | 0 (0) |
