# Supplementary material for: Self-application of aminoglycoside-based creams to treat cutaneous leishmaniasis in travelers
Source: PLoS Negl Trop Dis. 2023 Aug 10;17(8):e0011492. doi: 10.1371/journal.pntd.0011492 (PMC10443860; doi:10.1371/journal.pntd.0011492)
Supplement: S4 Table — (DOCX) [file pntd.0011492.s007.docx]

S4 Table: Listing of Serious Adverse Events

| Patient ID | Report Type | Gender | AE Description | Reason SAE | Start Date/ End Date |
| --- | --- | --- | --- | --- | --- |
| 1.03 | Initial | F | Pregnancy. Date of last menstrual period 24-MAR-2014. Pregnancy test (βHCG) negative at the date of 03-APR-2014 and positive the 28-APR-2014. Date of pregnancy estimated 10-APR-2014 and delivery the 24-DEC-2014. | Medically important condition | 110APR2014/ 24DEC2014 |
| 5.01 | Initial | M | Generalized papulo-nodular rash without pruritus. Concomitant to many chiggers's bites during a hunting session. | Medically important condition | 30SEP2015/ 10OCT2015 |
